# The Effect of Fathers’ Training Regarding Attachment Skills on Maternal-Fetal Attachments among Primigravida Women: A Randomized Controlled Trial

**Published:** 2014-10

**Authors:** Marzieh Akbarzade, Sara Setodeh, Farkhondeh Sharif, Najaf Zare

**Affiliations:** 1 Community Based Psychiatric Care Research Center, Department of Midwifery, School of Nursing and Midwifery, Shiraz University of Medical Sciences, Shiraz, Iran;; 2 Department of Midwifery, School of Nursing and Midwifery, Shiraz University of Medical Sciences, Shiraz, Iran;; 3 Community Based Psychiatric Care Research Center, Department of Mental Health and Psychiatric Nursing, School of Nursing and Midwifery, Shiraz University of Medical Sciences, Shiraz, Iran;; 4 Infertility Research Center, Department of Biostatistics, School of Medicine, Shiraz University of Medical Sciences, Shiraz, Iran

**Keywords:** Attachment, Father, Primigravida, Training

## Abstract

**Background:** Fathers’ cooperation has been less taken into account in the process of pregnancy. The present study aimed to investigate the effect of training the fathers regarding attachment skills on maternal-fetal attachment in primigravida women in 2013.

**Methods:** This randomized controlled trial was performed on 150 qualified pregnant women’s husbands. The intervention group took part in four 90-minute sessions of maternal-fetal attachment training held once a week. On the other hand, the control group received the routine pregnancy care. Both groups completed Spielberger’s anxiety scale and Cranley’s questionnaire before and after the intervention. Then, the data were analyzed using paired and independent t-test. Besides, P<0.05 was considered as statistically significant.

**Results:** The intervention group’s mean score of attachment was 55.98±6.99 and 61.90±5.41 before and after the intervention, respectively. The results of paired t-test revealed a significant difference between the intervention and the control group regarding their mean scores of attachment before and one month after the intervention (P<0.001). Additionally, the results of independent t-test showed a significant difference between the two groups regarding the five dimensions of the questionnaire, namely interaction with the baby (P<0.001), acceptance of maternal role (P<0.001), differentiation between oneself and the baby (P<0.001), attribution of some features to the baby (P=0.01), and self-devotion (P=0.01).

**Conclusion:** Training the fathers regarding the attachment behaviors and skills led to an increase in the maternal-fetal attachment scores. Thus, paternal training should be considered in pregnancy care programs.

**Trial Registration Number:** IRCT2012091910886N1

## Introduction

Having a baby is a great evolutionary change for the men who are experiencing being father for the first time. However, fathers’ emotions have been less taken into account in comparison to the mothers’ psychological changes. In general, pregnancy causes specific physiological changes in one’s sexual partner. In addition, labor and delivery are difficult-to-tolerate experiences for the fathers. Maternal health has been widely investigated by the medical staff for more than 80 years. However, fathers’ physical and mental health and how they can cooperate in the process of pregnancy have been less taken into account. In fact, fathering is an evolutionary stage for men.^1^Previously, a study was conducted on 53 English men whose wives were giving birth to their children through natural vaginal delivery and investigated the subjects for 60 hours after the delivery. The results showed that 57% of the men felt that they were under pressure, 56% were thinking about the pain their wives were tolerating at the moment, 38% believed that they were not effective in supporting their wives, and 22% were not interested in being present where their wives were giving birth to their children.^2^ Studies in the U.S., Britain, and other similar studies have reported fathers’ distress, unhappiness, and even disgust towards the infants during the first weeks of birth.^3^ In spite of the fathers’ positive expectations, some studies have shown than some fathers were unhappy rather than satisfied during the first months of their infants’ birth.^4^ Thus, more attention should be paid to the fathers’ readiness for accepting their roles. Besides, the role of fathers in improving the family’s health should be investigated, as well. In case the fathers are successful in sharing their wives’ experiences of pregnancy and creating more interaction with their infants, they will perform their paternal roles more desirably. According to studies, fathers’ cooperation in the pregnancy process is highly effective in their own, mothers’, infants’, and families’ health.^1^ The results of a study on the fathers expecting their children showed higher compatibility among the couples, lower anxiety, and higher paternal-fetal attachment among the fathers who had been trained for massaging their pregnant wives compared to the control group,^5^ which is consistent with the results of other studies conducted on the issue. Moreover, the findings of a study emphasized that paternal-fetal attachment before delivery was a prognostic factor for fetal attachment. In fact, paternal-fetal attachment was associated with paternal attachment during childhood, better outcomes of childhood adaptation, and improvement of family dynamics.^6^The men who shared their expectations with their wives and planned for their new roles showed more compatibility after the delivery in comparison to those who had not followed any specific plans.^5^

According to what was mentioned above, involving the pregnant women’s husbands in the training processes during pregnancy, labor, and delivery is of utmost importance for improving the paternal role as well as the emotions and relationships involved in pregnancy and having a child. However, most studies have focused on improvement of maternal-fetal attachment. Therefore, the present study aims to investigate the effect of training the fathers regarding attachment on the maternal-fetal attachment. 

## Materials and Methods

The present interventional randomized controlled trial aimed to determine the effect of training the fathers on maternal-fetal attachment. This study was approved by the Ethics Committee of Shiraz University of Medical Sciences. The research community included the primigravida women referring to the prenatal clinics of Hafez and Shoushtari hospitals, Shiraz, Iran. This study could not be blinded because of the interaction between the pregnant women and their husbands. Consequently, the subjects were selected through purposive sampling. According to the previous studies,^5^^,^^7^^-^^9^ considering α=0.05, 1-β=0.80, mean difference=1, and σ=3, and using the following formula, a 120-subject sample size was determined for the study.

$$n=\frac{{\left({\text{Z}}_{1-\frac{\alpha }{2}}+{\text{Z}}_{1-\beta }\right)}^{2}\sigma^{2}}{{\left(\mu_{1}-\mu_{2}\right)}^{2}}$$

**Figure 1:**
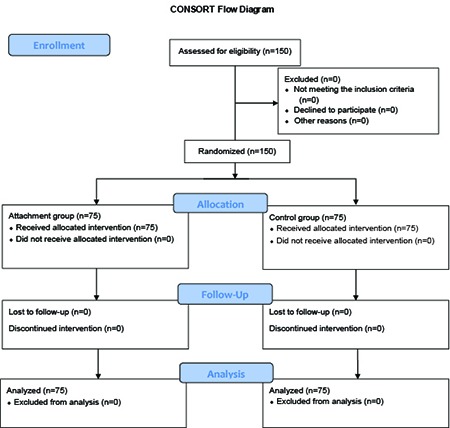
Study protocol consort diagram

However, considering the loss probability, 150 couples were selected through simple purposive sampling and were randomly divided into an intervention and a control group using the table of random numbers (figure 1). The inclusion criteria of the study for the mothers were being between 18 and 35 years old, single and planned pregnancy, gestational age of 28-34 weeks, not suffering from psychological disorders such as psychosis and schizophrenia, not suffering from chronic diseases such as cardiovascular disorders, pulmonary disorders, hypertension, and diabetes, having received the pregnancy care in the previous months, and having low or average anxiety levels according to Spielberger’s anxiety scale. In addition, the fathers’ inclusion criteria were having at least middle school degree, being below 45 years old, being able to take part in the training classes, and having signed written informed consents. On the other hand, the exclusion criteria of the study were being unwilling to cooperate in the study and incidence of any problems during pregnancy, such as abruption, cord prolapse, abnormal presentation, and placenta previa. It should be mentioned that cesarean delivery was not considered as an exclusion criterion.

In this study, Cranley’s questionnaire was utilized in order to assess the maternal-fetal attachment behaviors.^9^^,^^10^ This questionnaire evaluates maternal-fetal attachment behaviors in 5 dimensions, namely acceptance of maternal role (4 items), mutual interaction with the baby (5 items), attribution of some features to the baby (6 items), differentiation between oneself and the baby (4 items), and self-devotion (5 items). The study women were required to assign a score of 1-3 to each item. Therefore, the minimum and maximum scores of the questionnaire were 24 and 72, respectively. This questionnaire was translated into Persian by Khoramroudi and its validity was confirmed by 10 professors of School of Nursing and Midwifery of Iran University of Medical Sciences using content validity method. In addition, its reliability was assessed using test-retest method and confirmed by r=0.85. The reliability and validity indexes measured in Khoramroudi’s study are the basis of the present study.^11^

Spielberger’s anxiety scale was used in order to assess anxiety. This scale consists of 40 items, 20 of which assess state anxiety and the other 20 investigate trait anxiety. Accordingly, scores of 0-19, 20-40, 41-60, and 61-80 represent normal, mild, average, and severe anxiety, respectively. The sum of state and trait anxiety scores ranges from 20 to 80.^12^Aghamohammadi et al. used Spielberger’s scale in 150 patients and reported its reliability to be 97%. The reliability and validity indexes of the study by Aghamohammadi et al. are the basis of the current study.^13^

The study samples were selected using simple purposive sampling. In case the mothers had mild or average anxiety levels and their husbands met the inclusion criteria of the study, they were entered into the research. At first, the subjects were randomly divided into two groups through simple random sampling by flipping a coin. The intervention group underwent attachment training, while the other was considered as the control group. The pregnant mothers completed written informed consents, demographic questionnaires, and Cranley’s questionnaire both before and 1 month after the interventions. In the control group also, the questionnaires were completed at the same time as the intervention group. The interventions were performed during the 28^th^ to 34^th^ weeks of gestation. In doing so, the fathers were trained regarding the attachment skills through four 60-90-minute sessions held once a week. The contents of the sessions were as follows: first session: parental attachment to the baby and attachment behaviors, second session: concepts of maternal-fetal and paternal-fetal attachment and their effects on the parents’ physical and mental health and fetal growth, parents’ anxiety during pregnancy and its reasons and impacts, and father’s role in reduction of anxiety, third session: how attachment is created, beginning of attachment and its signs, father’s role, and acceptance of father’s role, and fourth session: father’s role in attachment, effect of focusing on the fetus, considering the fetus as an independent being, getting familiar with the sensory abilities of the fetus, and father’s role in breastfeeding after birth.

After the interventions, the fathers were followed up through telephone contacts and were asked to transfer their information to their wives. A reminder session was also held at the 38^th^ week of gestation.

After all, the mean scores of before and after the intervention were compared using paired t-test. Besides, independent t-test was used in order to compare the two groups. 

## Results

The mean age of the study women and their husbands was 24.59 and 29.03 years, respectively. The results of paired t-test showed no significant difference between the two groups regarding women’s and their husbands’ age distribution (P=0.218 and P=0.388, respectively) 

The results of independent t-test revealed no significant difference between the two groups regarding the total mean score of maternal-fetal attachment before the intervention (P=0.364). However, a significant difference was found in this regard 1 month after the intervention (P=0.001). Moreover, the results of paired t-test indicated a significant difference between the intervention group’s mean scores before and after the intervention (P<0.001), while no such difference was observed in the control group (P=0.660).

Before the intervention, no significant difference was found between the intervention and the control group regarding interaction with the baby (P=0.530), acceptance of maternal role (P=0.860), differentiation between oneself and the baby (P=0.770), attribution of features to the baby (P=0.210), and self-devotion (P=0.17). However, the results of independent t-test showed significant differences between the two groups regarding all the five dimensions one month after the intervention (P<0.05) (table 1).

**Table 1:** Mean and standard deviation of various aspects of maternal-fetal attachment before and after the intervention in control and attachment groups, Shiraz, 2012

**Dimensions of attachment behavior before the interventio**n		**Attachment**	**Control**	**P value**
		**Mean** **±** **SD**	**Mean** **±** **SD**	
Interaction with the baby	Before	10.57±1.19	10.69±1.13	0.530
	After	11.53±0.82	10.61±1.17	0.001
Acceptance of maternal role	Before	12.8±1.92	12.2±1.81	0.860
	After	13.13±1.69	12.04±1.81	0.001
Differentiation between oneself and the baby	Before	13.29±2.81	13.17±2.24	0.770
	After	14.69±2.35	13.33±2.20	0.001
Attribution of features to the baby	Before	9.49±1.61	9.81±1.52	0.210
	After	10.52±1.5	9.9±1.51	0.014
Self-devotion	Before	10.64±1.98	11.5±1.75	0.170
	After	11.81±1.84	10.97±2.17	0.012

Significance level: P≤0.05

## Discussion

The findings of the present study revealed a significant difference between the two groups regarding the total score of maternal-fetal attachment behaviors. The results of the study by Eun et al. showed no significant difference between the two groups regarding maternal-fetal attachment and level of anxiety before the intervention. After the intervention, however, the level of anxiety had decreased (P=0.08) and maternal-fetal attachment had increased (P=0.01) in the intervention group,^14^ which is in agreement with the results of the present study. However, the research communities of the two studies were different. In this study, the fathers took part in the classes and transferred the information to their pregnant wives.

Furthermore, a researcher investigated the effects of a training program (Taegyo) on parental attachment in primiparous women and their husbands. The couples of the intervention group underwent Taegyo for 4 weeks. After all, the findings indicated the effectiveness of the training program in increasing the parental as well as maternal-fetal attachment in the first pregnancy.^15^These results were in line with those of the present study.

Moreover, a researcher conducted a study on 442 pregnant women (divided into 3 groups) in a hospital in Kathmandu, Nepal in order to investigate the effect of husbands’ presence in training classes on women’s awareness and mental health. In the first group, both men and women took part in the training classes, while the second group included the women who participated in the classes without their husbands. Finally, the women in the control group underwent no interventions. According to the results, the first group showed higher awareness scores and a 0.61-point increase in mental health (P<0.001), while only a 0.34-point increase was observed in the second group’s scores (P<0.05). Overall, a significant difference was observed between the intervention groups and the control group regarding mental health and increase of awareness.^16^

The findings of the current study revealed a significant difference between the intervention and control groups with respect to the five dimensions of Cranly’s questionnaire (P≤0.05). Yet, the highest scores were related to “differentiation between oneself and the baby” and “acceptance of maternal role” domains, while the lowest scores were related to “interaction with the baby” and “attribution of features to the baby”, which is consistent with the results of other investigations.^5^^,^^17^ The lower scores in these two domains might be due to various types of interactions between the mother and the baby, including talking with the baby, fetal abdominal palpation, and reading poems and stories. These behaviors often occur when a fetus is not still considered as an independent being by the parents. Of course, this may not be acceptable for all the mothers since they think that the babies do not hear their voice. Another possible factor may be the mother’s fear of harming the child or being shameful for doing these behaviors.

In the current study, a significant difference was found between the two groups regarding the first dimension of the questionnaire; i.e., interaction with the fetus, one month after the intervention (P<0.001). The items of this dimension were: “I talk with my baby”, “I think that the baby wants to remind me of the eating time by moving”, etc. This shows the effectiveness of the attachment trainings concerning taking with the fetus, paying attention to its movements, and father’s cooperation in interaction with the mother and the fetus.

The findings of independent t-test also showed a significant difference between the two groups regarding the second dimension of the questionnaire; i.e., acceptance of maternal role (P<0.001). This dimension included items such as “I enjoy watching my abdomen moving”, “I assume myself breastfeeding the baby”, etc. By training the fathers to be involved in breastfeeding, changing their assumption of being inactive and inefficient towards a father who plays a critical role in breastfeeding and hugging the baby, and sharing their emotions with their wives, the intervention was effective in mothers’ acceptance of their roles, paying more attention to the baby, considering the baby as an independent being, and assuming her future interactions with the baby.  

Regarding the third dimension (differentiation between oneself and the baby), the study results revealed a significant difference between the intervention and the control group (P<0.001). The items of this dimension included “I am interested to know if my baby hears the sounds”, “I do not know whether my baby can feel anything”, etc. Training the fathers regarding interaction with the baby as an independent being which can identify sounds and sharing their perceptions of this new being with the mother caused the mothers to consider the baby as an independent being. Thus, the researcher was successful in providing the ground for more interaction between the parents and the fetus.

The study findings also showed a significant difference between the two groups concerning the fourth dimension of the questionnaire (P=0.014). The items of this dimension were: “I try to assume what my baby looks like”, “I have decided on boy’s name”, “I have decided on girl’s name”, etc. It should be noted that this dimension mostly depends on the mother’s assumption and training the fathers can be effective in mentally preparing the mothers. Thus, by emphasizing the importance of the above-mentioned questions to the fathers and asking them to transfer their imaginations to the mothers, the researcher created more enthusiasm in the parents which eventually increased the mothers’ attachment.

Finally, the findings of the current study indicated a significant difference between the two groups regarding the fifth dimension of the questionnaire; i.e., self-devotion (P=0.012). The items of this dimension included “I do not do some tasks so that my baby will be comfortable”, “I feel that my body is ugly”, etc. This dimension mostly dealt with mothers’ devotion and maternal feelings which are strengthened by a trained father. It seems that the educational intervention of the present study was effective in this regard.

Research has shown that the men who shared their parental expectations and new roles with their wives could cope with postpartum processes more easily compared to those who did not.^5^ Also, a 3-fold decrease was observed in maternal anxiety during pregnancy.^18^

Another study examined the effect of education on maternal–fetal attachment behavior, using Cranly’s questionnaire for assessment of maternal–fetal attachment behaviors. In that study, mothers noted their babies’ movements during classes. The results indicated an increase in various aspects of maternal-fetal attachment in the intervention group compared to the control group.^19^ Similar results were also obtained in another study conducted on the issue.^20^ The difference between these studies and the present one is that the above-mentioned studies only trained the mothers, while fathers were trained in our study. The researchers could find no studies with opposite results with those of the current study within the last 20 years.

Overall, it can be concluded that training the fathers and involving them in the process of pregnancy and attachment were effective in all the attachment dimensions and enhanced the maternal-fetal attachment. When a couple is going to have a baby, they experience a large number of changes. This necessitates informing the young fathers regarding the new roles, plans, and relationships in this transitional stage. Yet, this major stage can be slightly simplified by having realistic expectations and having information about the requirements of this stage. In fact, one of the great wonders of fathering is the change in the marital relationship after having a child.^21^Since the quality of maternal-fetal attachment is different from that of paternal-fetal attachment, the very first hours after birth are quite sensitive for creation of maternal-fetal attachment. Moreover, fathers’ cooperation can result in safe attachments and the more time the fathers spend with their children, the stronger this effect will be.^22^

There are several studies to report effective interventional programs for development of maternal-fetal attachment, including the study Carter performed on fetal palpation^23^ and the one Brayan conducted on training the couples and increasing their awareness about infant care.^24^ Moreover, Schachman et al. trained stress management method^25^ and Chang et al. suggested expressing affection to the fetus by writing letters and talking to the fetus.^26^

In these studies, maternal attachment to the fetus could be enhanced by sensitivity of the maternal perception of fetal movements and training was provided for the mothers. In the present study, training was given to the father, and then the father was required to transfer the educational material to his pregnant wife.

This method led to fathers’ cooperation and improved the mothers’ maternal-fetal relationships.

One of the strong points of this project was fathers’ participation in their pregnant wives’ pregnancy care process. This created a sense of responsibility in the husbands for transferring the information to their pregnant wives. However, one of the limitations of the study was the husbands’ difficulty in expressing their wives’ problems in the presence of other male participants. Another limitation of this research was that it could not be blinded. Moreover, further studies are recommended to involve both mothers and fathers in maternal-fetal attachment training classes. 

## Conclusion

According to the present study findings, training the fathers regarding the attachment skills and transferring them to their wives enhanced the maternal-fetal attachment. Thus, it seems that teaching the attachment behaviors to fathers, as a new obstetric training, can increase the maternal-fetal attachment. Hence, midwives, nurses, and other health staff who are in more contact with the couples are recommended to teach this simple, inexpensive, and enjoyable technique to the parents as a part of prenatal care services. In this way, steps can be taken towards improvement of maternal as well as fetal health. Yet, further studies are recommended to be blinded and consider a different intervention for the control group participants. 

## References

[1] Bartlett EE (2004). The effects of fatherhood on the health of men: a review of the literature. The Journal of Men’s Health & Gender.

[2] Johnson MP (2002). An exploration of men’s experience and role at childbirth. J Men’s Studies.

[3] Redshaw M, Henderson J (2013). Fathers’ engagement in pregnancy and childbirth: evidence from a national survey. BMC Pregnancy Childbirth.

[4] Buist A, Morse CA, Durkin S (2003). Men’s adjustment to fatherhood: implications for obstetric health care. J Obstet Gynecol Neonatal Nurs.

[5] Latifses V, Estroff DB, Field T, Bush JP (2005). Fathers massaging and relaxing their pregnant wives lowered anxiety and facilitated marital adjustment. Journal of Bodywork and Movement Therapies.

[6] Wilson ME, White MA, Cobb B (2000). Family dynamics, parental-fetal attachment and infant temperament. Journal of Advanced Nursing.

[7] Bryan AA (2000). Enhancing parent-child interaction with a prenatal couple intervention. MCN Am J Matern Child Nurs.

[8] Bellieni CV, Ceccarelli D, Rossi F (2007). Is prenatal bonding enhanced by prenatal education courses?. Minerva Ginecol.

[9] Ustunsoz A, Guvenc G, Akyuz A, Oflaz F (2010). Comparison of maternal-and paternal-fetal attachment in Turkish couples. Midwifery.

[10] Cranley MS (1981). Development of a tool for the measurement of maternal attachment during pregnancy. Nurs Res.

[11] Khoramrody R (2000). The effect of mothers touch on maternal fetal attachment [thesis].

[12] Adler J, Urech C, Finc N (2011). Response to Induced Relaxation During Pregnancy: Comparison of Women with High Versus Low Levels of Anxiety. Journal of Clinical Psychological Medicine Setting.

[13] Kalkhoran MA, Karimollahi M (2007). Religious and preoperative anxiety: a correlational study. Annals of General Psychiatry.

[14] Ji ES, Han HR (2010). The effect of Qi exercise on maternal-infant interaction and maternal well being during pregnancy. J Obstet Gynecol Neonatal Nurs.

[15] Yang KM, Kim SL (2010). Effects of a Taegyo Program on Parent-Fetal Attachment and Parenthood in First Pregnancy Couples. J Korean AcadNurs.

[16] Mullany BC, Lakhey B, Shrestha D (2009). Impact of husbands’ participation in antenatal health education services on maternal health knowledge. JNMA J Nepal Med Assoc.

[17] Levine A, Zagoory-Sharon O, Feldman R, Weller A (2007). Oxytocin during pregnancy and early postpartum: individual patterns and maternal-fetal attachment. Peptides.

[18] Toosi M, Akbarzadeh M, Sharif F, Zare N (2014). The Reduction of Anxiety and Improved Maternal Attachment to Fetuses and Neonates by Relaxation Training in Primigravida Women. Women’s Health Bulletin.

[19] Bellieni CV, Ceccarelli D, Rossi F (2007). Is prenatal bonding enhanced by prenatal education courses?. Minerva Ginecol.

[20] Mikhail MS, Freda MC, Merkatz RB (1991). The effect of fetal movement counting on maternal attachment to fetus. Am J Obstet Gynecol.

[21] Ramchandani P, Stein A, Evans J (2005). Paternal depression in the postnatal period and child development: a prospective population study. Lancet.

[22] Saisto T, Salmela-Aro K, Nurmi JE, Halmesmaki E (2001). Psychosocial characteristics of women and their partners fearing vaginal childbirth. British Journal of Obstetrics and Gynaecology.

[23] Carter-Jessop L (1981). Promoting maternal attachment through prenatal intervention. MCN Am J Matern Child Nurs.

[24] Bryan AA (2000). Enhancing parent-child interaction with a prenatal couple intervention. MCN Am J Matern Child Nurs.

[25] Schachman KA, Lee RK, Lederma RP (2004). Baby boot camp: facilitating maternal role adaptation among military wives. Nurs Res.

[26] Chang S, Park S, Chung C (2004). Effect of Taegyo-focused prenatal education on maternal-fetal attachment and self-efficacy related to childbirth. Taehan Kanho Hakhoe Chi.

